# Successful outcome following pneumonectomy in a teenage boy with cystic fibrosis: a case report

**DOI:** 10.1186/s12890-016-0350-x

**Published:** 2017-01-13

**Authors:** Zheyi Liew, Santosh Mallikarjuna, Asif Hasan, F. Kate Gould, Su Bunn, Matthew F. Thomas, Jim L. Lordan, Christopher O’Brien, Malcolm Brodlie

**Affiliations:** 1 Department of Paediatric Respiratory Medicine, Great North Children’s Hospital, Newcastle upon Tyne, UK; 2 Department of Paediatric Cardiothoracic Surgery, Freeman Hospital, Newcastle upon Tyne, UK; 3 Institute of Transplantation, Freeman Hospital, Newcastle upon Tyne, UK; 4 Department of Paediatric Gastroenterology, Great North Children’s Hospital, Newcastle upon Tyne, UK; 5 Institute of Cellular Medicine, Newcastle University and Department of Paediatric Respiratory Medicine, Great North Children’s Hospital, Level 3 Clinical Resource Building, Royal Victoria Infirmary, Queen Victoria Road, Newcastle upon Tyne, NE1 4LP UK

**Keywords:** Cystic fibrosis, Pneumonectomy, Destroyed lung, Paediatrics, Case report

## Abstract

**Background:**

Cystic fibrosis lung disease is generally a diffuse process however rarely one lung may become particularly damaged through chronic collapse and consolidation resulting in end-stage bronchiectasis with relative sparing of the contralateral lung. This clinical situation is sometimes referred to as “destroyed lung”. Lung resection surgery is seldom indicated in cystic fibrosis and the associated medical literature is relatively sparse.

**Case presentation:**

A 14 year old boy was referred to our centre for lung transplantation assessment. He had a chronic history of complete collapse and consolidation of his entire right lung. This was causing severe morbidity in terms of a continuous requirement for intravenous antibiotics over the last year, poor exercise tolerance with forced expiratory volume in 1 s of 35–40% predicted and need for home tuition. He also had significant nutritional problems and gastrointestinal symptoms following a Nissen’s fundoplication operation a year earlier. His nutritional status was firstly improved by the institution of jejunal feeding, which also greatly improved his distressing symptoms of nausea and wretching. After thorough multidisciplinary assessment the therapeutic option of performing a right pneumonectomy was considered due to relative sparing of the left lung, which demonstrated only mild bronchiectasis on computed tomography scan. This was performed uneventfully with a smooth peri-operative course. Targeted antimicrobials were used to treat the multiresistant organisms colonising his airways. Subsequently his quality of life, nutritional status and lung function all improved significantly and requirement for lung transplantation has been delayed.

**Conclusions:**

We report a successful outcome following pneumonectomy in a teenage boy with cystic fibrosis referred to our centre for lung transplantation assessment with chronic unilateral collapse and consolidation of his right lung. We believe that improvement of nutritional status pre-operatively and targeted antimicrobial therapy, all contributed to the smooth peri-operative course. Pneumonectomy can be a feasible option in this clinical situation in cystic fibrosis but the associated risks must be considered carefully on a case-by-case basis.

## Background

Cystic fibrosis (CF) is the most common genetically acquired life-limiting disorder in populations of European ancestry. Lung disease is responsible for the vast majority of morbidity and mortality in people with CF [1]. Lung transplantation is the only therapeutic option for end-stage disease but is itself associated with morbidity and limited survival. CF lung disease is characterised by progressive neutrophilic airway inflamation, retention of mucopurulent secretions and endobronchial infection with specific microbes, most notably *Pseudomonas aeruginosa* [2].

CF lung disease is generally diffuse and bilateral although inhomogeneity is recognised between different lobes and rarely disease may become markedly severe in specific lobes or in one lung [3, 4]. If a collapsed/consolidated lung is not reinflated then a vicious cycle of mucus impaction, infection and inflammation can lead to irreversible damage [5, 6]. In extreme cases a phenomenon known as ‘destroyed lung’ may occur where an entire lung is irreversibly damaged, contributing little to ventilation yet causing significant problems with chronic infection [6]. Potentially life-threatening complications may occur including septicaemia, haemoptysis, empyema, pulmonary-systemic shunting and pulmonary hypertension [5, 6]. Surgical pneumonectomy to remove the destroyed lung may be indicated but is not without associated risks and importantly is only a viable option if the contralateral lung is not severely involved. On occasions one specific lobe may be causing severe problems and consideration may be given to the pros and cons of lobectomy in such cases [7, 8].

Published literature on lung resection in children with ‘destroyed lung’ in general is relatively limited with even fewer examples in children specifically with CF. In 2003, Eren et al. reported the results of surgery in a series of 17 children undergoing pneumonectomy for ‘destroyed lung’ secondary to suppurative or necrotic lung disease, but not CF [5]. In this series two children died in the peri-operative period and four developed post-operative complications including empyema, haemorrhage requiring re-thoracotomy and broncho-pleural fistula. Longer-term follow up suggested that the majority of surviving children had increased exercise tolerance [5]. In 2010 Kosar et al. reported comparatively improved outcomes in a non-CF case series of 18 children who underwent pneumonectomy for ‘destroyed lung’ [6]. Pre-operative ventilation-perfusion scans demonstrated that contribution from affected lungs was minimal. In this series there was no peri-operative mortality and a complication rate of 17% including a wound infection and bronchopleural fistula and empyema. The authors concluded that favourable outcomes were due to case selection, optimisation of nutritional status, use of targeted antimicrobials and pro-active physiotherapy post-operatively [6]. Smith et al. also reported their experience of nine paediatric and four adult patients with CF who underwent a total of 17 major lung resections over a decade in the 1980’s [9]. Three pneumonectomies were performed in children. Overall in 75% of the pneumonectomy patients there was a decrease in symptoms, increased sense of well-being, and reduction in admission rates post-operatively [9].

In this paper we report a successful outcome following pneumonectomy in a teenager with a ‘destroyed’ right lung referred to our centre for lung transplantation assessment.

## Case presentation

**Fig. 1:**
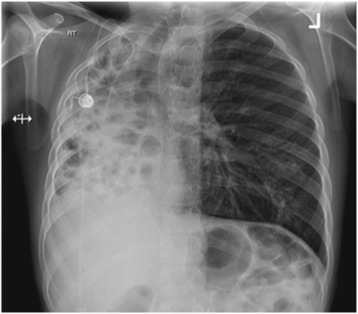
Pre-operative chest radiograph at time of initial assessment showing presence of severe *right* lung collapse and consolidation leading to mediastinal shift to the right with hyperexpansion of the *left* lung

**Fig. 2:**
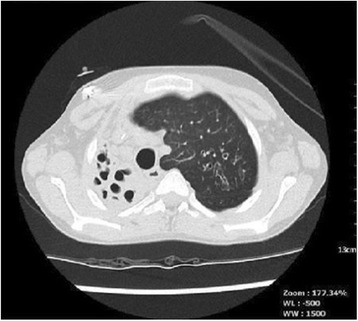
Pre-operative chest high-resolution computed tomography scan showing severe bronchiectasis of the right lung and hyper-expansion of the left lung with mediastinal shift to the right, but relatively minor bronchectasis

A 14-year-old boy with CF (*CFTR* genotype *Phe508del/Phe508del*) was referred for lung transplant assessment. He was diagnosed with CF as a 3 month old infant having presented with recurrent pneumonia and malabsorption. His forced expiratory volume in 1 s (FEV_1_) was 0.8 L (39% predicted) and there was a severe problem with collapse and consolidation of his entire right lung (Fig. 1). He completed 395 m with lowest oxygen saturations of 92% during a 6 min walk test in room air. His lung function had progressively reduced over the previous 4 years with chronic collapse of the right lung evident on chest radiographs for the last 2 years. He was experiencing significant morbidity in association with these problems including a continuous requirement for intravenous antibiotics over the preceding 12 months, limited exercise tolerance necessitating home tuition and overall poor quality of life. As a consequence his left lung was hyper-expanded but notably had only mild bronchiectasis on a recent high-resolution computed tomography scan (Fig. 2). Pan-resistant *Pseudomonas aeruginosa* and *Stenotrophomonas maltophilia* were commonly isolated from his sputum along with *Aspergillus terreus*. Non-tuberculous mycobacteria nor *Mycobacterium tuberculosis* had ever been cultured from respiratory samples.

The boy also had substantial gastrointestinal and nutritional problems. He had undergone a Nissen’s fundoplication around a year previously at his home centre to try and improve problems with gastro-oesophageal reflux. However he had continued to experience distressing symptoms of nausea, retching and vomiting, particularly in association with gastrostomy feeds, that limited his nutritional intake post-fundoplication. At referral his weight at 26.6 kg and height at 1.39 m, were both significantly below the 0.4th centiles for his age.

An inpatient assessment was arranged in our centre during which a trial of stopping intravenous antibiotics led to rapid signs of sepsis. His debilitating gastrointestinal problems were also clearly evident and he was also reviewed by a Consultant Paediatric Gastroenterologist. His abdominal symptoms were felt to be suggestive of gastroparesis or delayed gastric emptying and visceral hypersensitivity. He was started on gabapentin for visceral hypersensitivity and plans were made for a further admission for a trial of jejunal feeding using an amino acid feed to further manage symptoms and improve nutritional status.

In parallel careful multidisciplinary discussions were held and although relatively high risk it was felt that a right pneumonectomy could be a beneficial intervention to remove a chronic sump of infection and improve his current status as a strategy to delay the need for active listing for lung transplantation. It was confirmed that implantation of a single donor lung, rather than bilateral lung transplantation, was the only viable option at any stage in his case due to chronic remodelling that had occurred of the right hemithorax with markedly reduced volume compared to the left. This was discussed fully with the patient and his family and a plan was agreed to improve his nutrition as far as possible prior to performing a pneumonectomy.

He was subsequently re-admitted and a jejunal extension was added to his gastrostomy and a flexible bronchosocopy performed. The bronchoscopy revealed normal left-sided bronchial anatomy and on the right a tracheal bronchus with mucoid plugs obstructing all major bronchi. Bronchoalveolar lavage fluid grew *S. maltophilia, A. Terreus* and *Staphylococcus aureus*. Continuous jejunal feeding (Elemental E208, Nutricia) was introduced and led to a rapid gastrointestinal symptomatic improvement with much less retching and vomiting.

Following three weeks of jejunal feeding, during which time he gained 2.4 kg, the pneumonectomy was performed. The operation was surgically uneventful, he was extubated within 24 h and was discharged from intensive care to the ward on the first post-operative day. Amikacin, piperacillin/tazobactam, moxifloxacin and posaconazole were used to provide targeted antimicrobial cover. His subsequent course was smooth with rapid improvement in chest symptoms and successful withdrawal of intravenous antibiotics. His gastrointestinal symptoms also continued to improve and he gained a further 1 kg prior to discharge home three weeks later.

**Fig. 3:**
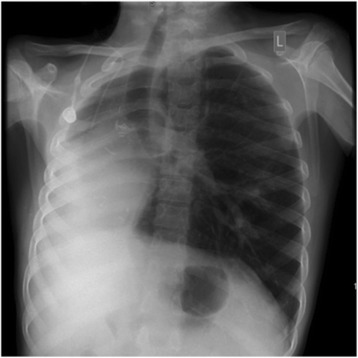
Chest radiograph around 6 months post-pneumonectomy

In the 2 years following surgery he has been followed up by his local centre. There has been a dramatic improvement in quality of life, evidenced by good school attendance, physical activity and that he has only required admission to hospital for one course of intravenous antibiotics. Importantly his lung function has increased substantially to around 65–70% predicted FEV_1_ and he has gained a further 5 kg in weight with now both height and weight following the same centile. Figure 3 shows a chest radiograph around 6 months post-pneumonectomy.

## Conclusions

We report a successful outcome following pneumonectomy in a teenage boy with CF and ‘destroyed’ right lung as a strategy to improve his clinical status and quality of life to an extent that it has delayed requirement for active lung transplantation listing. Pneumonectomy is not without associated risks and is an uncommon procedure in CF as it is generally a diffuse lung disease. This case demonstrates however that it can be a feasible option in this extreme clinical situation.

We believe that careful case selection, improvement of nutritional status pre-operatively and targeted antimicrobial therapy all contributed to the smooth peri-operative course and successful outcome. All of these factors must be balanced against the risks of surgery and pneumonectomy should only be considered in patients with severe unilateral problems. Furthermore, post-pneumonectomy children may develop problems with scoliosis that may be severe and associated with respiratory problems. In general optimal timing of surgery with regard to the pubertal growth spurt and consideration of the use of prophylactic tissue expanders may reduce the likelihood of these issues however [10].

This intervention has improved the patient’s quality of life considerably - both lung function and weight have increased substantially. It should be noted that in the context of a previous contralateral pneumonectomy future single lung transplantation may be technically more challenging but ultimately has been associated with good outcomes [11, 12].

The boy in this case experienced significant gastrointestinal symptoms, in the form of gastroesophageal reflux disease pre-fundoplication, and severe problems post-fundoplication likely secondary to gastrointestinal dysmotility. The exact relationship between gastroesophageal reflux and aspiration of refluxate in CF lung disease is unclear but there is potential for this to contribute to airway injury and reflux-induced cough and reported symptoms have been associated with reduced lung function in adults [13–15]. It is uncertain as to how he developed such severe problems in the right lung but it is possible to speculate that recurrent reflux/aspiration may have played some role pre-fundoplication. In addition the presence of a tracheal bronchus was of uncertain contribution to the development of his problems on the right side. Histology of the resected lung showed severe bronchiectasis throughout all lobes with a dense inflammatory infiltrate of neutrophils and lymphocytes. In general we would recommend a proactive approach in any child or young person with CF that develops lobar collapse and consolidation. This is likely to involve bronchoscopic clearance of mucus and secretions, targeted antimicrobials, mucolytics and intensive physiotherapy in order to try and avoid more chronic problems developing and irreversible lung damage.

## Abbreviations

CF: Cystic fibrosis
FEV_1_: Forced expiratory volume in 1 s
